# Baicalin-copper complex alleviates intestinal damage in avian pathogenic *Escherichia coli*-infected chicks by targeting the AKT/NF-κB pathway

**DOI:** 10.3389/fvets.2025.1648736

**Published:** 2025-08-26

**Authors:** Panpan Cao, Jiazhen Wei, Xinyi Cheng, Yu Zhuang, Junrong Luo, Huabin Cao, Caiying Zhang, Xiaoquan Guo, Guoliang Hu

**Affiliations:** ^1^Jiangxi Provincial Key Laboratory for Animal Health, College of Animal Science and Technology, Institute of Animal Population Health, Jiangxi Agricultural University, Nanchang, China; ^2^School of Jiangxi Biological Vocational College, Nanchang, China

**Keywords:** baicalin-copper complex, avian pathogenic *Escherichia coli*, intestinal, chicks, AKT/NF-κB

## Abstract

**Background:**

Avian pathogenic *Escherichia coli* (APEC), a primary bacterial pathogen in poultry, induces substantial economic losses to the global poultry industry. The baicalin-copper complex (BCU) demonstrates markedly potentiated anti-inflammatory, anti-oxidant, and anti-tumor efficacy relative to either baicalin or copper in their isolated forms. The present study aimed to evaluate the protective effects of BCU against APEC-induced intestinal damage in chicks.

**Methods:**

Seventy-five one-day-old chicks were randomly assigned to five groups: control group (basic diet), *E. coli* group (basic diet), and BCU treatment groups (10, 20, and 40mg/kg BCU). After a 15-day feeding period, APEC infection was induced via pectoralis injection to ensure consistent systemic infection. Two days later, the chicks were weighed, and blood samples from the pterygoid vein and ileum tissue were collected for subsequent experiments.

**Results:**

The results showed that compared with the *E. coli* group, BCU reduced both diarrhea and mortality rates, with reductions in the BCU40 group to 27% and 7%, respectively. It also significantly up-regulated the mRNA expression of key intestinal physical barrier proteins (ZO-1, ZO-2, Claudin-1, Claudin-3, and Occludin) and chemical barrier components (Mucin 2 (MUC2) and avian β-defensins (AvBD2, AvBD4) (*p* < 0.05). Compared with the *E. coli* group, as shown by BCU markedly increased activities of antioxidant enzymes GSH-Px, CAT, SOD and reduced MDA level, which along with increased mRNA expression of the Nrf2-antioxidant signaling pathway (*p* < 0.05). Furthermore, BCU significantly down-regulated the mRNA expression of pro-inflammatory cytokines TNF-α, IL-1β, IL-6, IL-8, and significantly up-regulated the anti-inflammatory cytokines IL-10 and TGF-β (*p* < 0.05). Moreover, BCU inhibited the AKT/NF-κB signal pathway, as indicated by markedly reduced the protein expression of p-NF-κB and p-AKT (*p* < 0.01).

**Conclusion:**

Collectively, the findings suggested that BCU effectively alleviates intestinal damage induced by APEC-infection through AKT/NF-κB signal pathway to modulate oxidative stress and inflammatory response.

## Introduction

Avian pathogenic *Escherichia coli* (APEC) is a major bacterial pathogen responsible for colibacillosis in poultry, particularly affecting young chicks. This pathogen induces diarrhea, intestinal hemorrhage, and systemic inflammation, elevating mortality rates while severely compromising growth performance—imposing substantial economic burdens on global poultry production (1). APEC’s virulence targets intestinal barrier integrity, provoking dysregulated immune responses. Current reliance on antibiotic-based control strategies exacerbates antimicrobial resistance risks, necessitating natural alternative interventions.

Intestine is the main target organ of APEC, which consists of physical barriers, chemical barriers, and biological barriers, comprising tight junction proteins (e.g., ZO-1, claudins, occludin) and mucosal immune molecules such as mucin 2 (MUC2) and avian β-defensins (AvBD2, AvBD4), plays a pivotal role in host defense. The destruction of intestinal barrier will induce the inflammatory reaction of the body. Nuclear factor Kappα-B (NF-κB) is a unique transcription factor that can be activated by various pathological stimuli, has a key role in regulating the expression of numerous inflammatory factor genes, and serves as an essential component for the transcription of multiple pro-inflammatory genes. APEC infection disrupts barrier, exacerbating oxidative stress and inflammatory damage through the activation of signaling pathways such as AKT/NF-κB (2). Excessive activation of NF-κB promotes the expression of pro-inflammatory cytokines and oxidative mediators, creating a vicious cycle of tissue injury (3).

Baicalin, a flavonoid compound extracted from *Scutellaria baicalensis*, has demonstrated a wide range of biological effects, including anti-inflammatory, anti-oxidant, and anti-bacterial properties (4). Copper, an essential trace element, plays vital roles in immunomodulation, antioxidant defense, and tissue homeostasis (5). Critically, the baicalin-copper complex (BCU) demonstrates synergistically enhanced bioactivity compared to either component alone, BCU administration improves intestinal health, suppresses inflammatory cascades, and enhances growth metrics in avian models (6). It also possesses strong antimicrobial activity against pathogens such as *E. coli*, *Staphylococcus aureus*, and *Candida albicans* (7), and can inhibit tumor cell proliferation *in vitro* (8).

Plant-derived bioactive compounds have shown great promise in modulating gut microbiota, immune function, and antioxidative capacity in livestock. For instance, *Litsea cubeba* essential oil improved antioxidant status, nutrient digestibility, and immune responses in pigs (9). Similarly, shifts in gut microbiota have been linked to immune regulation and disease progression in various host systems (10), collectively supporting BCU’s potential as a multifunctional phytogenic additive. However, few studies have explored the protective mechanism of BCU against APEC-induced intestinal damage in poultry. Building on BCU’s documented pharmacological versatility and microbiota-metabolite regulatory capacity, the present study utilized 75 one-day-old chicks to evaluate the effects of dietary BCU on intestinal integrity, oxidative stress, and inflammatory responses, and AKT/NF-κB signaling modulation in APEC-challenged chicks. These findings advance novel phytochemical-based strategies for poultry health enhancement.

## Materials and methods

### The sources of BCU and avian pathogenic *Escherichia coli*

BCU in the experiment was obtained from Xing ding technology biotechnology Company (China), and *Escherichia coli* APAP-O78 was procured from the China Institute of Veterinary Drug Control (#CVCC1418).

### Animals, diets, and management

Seventy-five chicks aged 1-day-old Hy-line brown laying hens were maintained under protocols approved by Jiangxi Agricultural University Animal Care and Use Committee. Chicks were housed in a controlled environment with standardized temperature, humidity, and lighting. After 7-day acclimation, the chicks were allocated to five treatment groups: control (basal diet), *E. coli* (basal diet + APEC challenge), BCU10 (basal diet + 10 mg BCU/kg), BCU20 (basal diet + 20 mg BCU/kg), and BCU40 (basal diet + 40 mg BCU/kg), with 15 chicks per group. The composition of the diets are shown in Table 1. On day 15 of the experiment, chicks in all groups except the control group were intramuscularly injected with 0.5 mL of *E. coli* suspension (3.39 × 10^9^ CFU/mL) into the pectoralis major muscle. Intramuscular injection can effectively ensure the establishment of systemic infection. The control group received an equal volume of sterile saline. After 2 days, the chicks were weighed, and wing vein blood samples were collected. All chicks were then anesthetized via intravenous injection of sodium pentobarbital (50 mg/kg). Ileum tissues were snap-frozen in liquid nitrogen and stored at −80 °C for molecular analysis.

**Table 1:** Composition and nutrients levels of diets %.

Dietary composition %	Content %
Corn	66.00
Soybean meal	29.00
Premix^a^	5.00
Total	100.00
Nutrient level	
DL-Methionine %	0.26
Lysine %	0.88
Metabolic energy (MJ/kg)	11.92
Crude protein %	17.61

a Premix: copper: 8 g; iron: 67 g; zinc: 87 g; cobalt: 94 g; sodium selenite: 11.25 g; potassium iodide: 8.75 g; DL-methionine: 300 g; polysaccharide vitamin: 100 g; sodium chloride: 2,000 g; mountain flour: 13,000 g; calcium hydrogen phosphate: 6,000 g.

### Record the diarrhea and mortality rates

After the injection of *E. coli* liquid, diarrheal incidence and mortality were recorded daily, and the diarrhea and mortality rates were counted as cumulative deaths per group.

### Morphology observation

According to the method described by Tang et al. (7), Hematoxylin–Eosin (H&E) staining method was used for observation. Three centimeters of ileal tissue was cut, and the inner wall of the intestine was gently flushed with saline, the operation process should be avoided to damage the intestinal mucosa as much as possible. Finally, tissue sections were made and observed with an optical microscope (Olympus BX63, 40 × 10).

### Determination of oxidative stress indexes

Following the methods reported by Luc et al. (8), the optimal sampling concentrations for each assay and each sample were explored by UV spectrophotometry according to the diagnostic kit manufacturer’s instructions (correlation coefficient: *r* = 0.99, confidence level: *p* < 0.001, Nanjing Jiancheng Bio, China). Experiments were performed based on the results of the optimal sampling concentrations from the above experiments. Superoxide dismutase (SOD), catalase (CAT), glutathione peroxidase (GSH-Px) activities and malondialdehyde (MDA) levels were determined strictly according to the kit instructions.

### Real-time quantitative PCR analysis (q-PCR)

Total RNA was extracted from ileal tissues using standard protocols. Reverse transcription was performed to synthesize complementary DNA (cDNA). q-PCR was carried out as detailed in the previous paper (11). The expression levels of genes including TNF-α, interleukins (IL-1β, IL-6, IL-8, IL-10), transforming growth factor-β (TGF-β), tight junction proteins (ZO-1, ZO-2), claudins (Claudin1, Claudin3), occludin, mucin 2 (MUC2), avian β-defensins (AvBD2, AvBD4), nuclear respiratory factor 2 (Nrf2), heme oxygenase-1 (HO-1), CAT, SOD-1, glutathione peroxidase 1 (GPX1), and glutamate-cysteine ligase modifying subunits (GCLM) were analyzed. Q-PCR was conducted using specific primers in Table 2. GAPDH was selected as the internal control (housekeeping gene) to normalize target gene expression. All primers were verified for specificity by melting curve analysis, and efficiency was within acceptable range (90–110%). The relative mRNA expression levels were quantified using the comparative Ct method (2^−ΔΔCt^).

**Table 2:** Sequence of target genes primer.

Gene names	Primer sequence (5′–3′)
TNF-α	F: CAGATGGGAAGGGAATGAAC
	R: CACACGACAGCCAAGTCAAC
IL-1β	F: GGTCAACATCGCCACCTACA
	R: CATACGAGATGGAAACCAGCAA
IL-6	F: GCCAGAGCCAGGGAGAATATC
	R: CCCTCACGGTCTTCTCCATAAA
IL-8	F: GCAAGGTAGGACGCTGGTAA
	R: GCGTCAGCTTCACATCTTGA
IL-10	F: GGTTGTCGTCTCATTCTGAAAGA
	R: GGTAGAGGACCCAAGTTCGTTAAGA
TGF-β	F: GCTCCACGGAGAAGAACAGGCTG
	R: CTGCTCCACCTTGGGCTTGC
ZO-1	F: GGCAGCTATCAGACCACTCT
	R: ACTTGTAGCACCATCTGCCT
ZO-2	F: CGGACTGTCATCTCGTTCAGGCAC
	R: GCTGGGAAGGAAGAGAACCT
Claudin 1	F: TACAGCCCTTGGCCAATACA
	R: CCAAGAAACAACCACCAGCA
Claudin 3	F: AAGGTGTACGACTCCATGCT
	R: CGATGGTGATCTTGGCCTTG
Occludin	F: CCTCATCGTCATCCTGCTCT
	R: GGTCCCAGTAGATGTTGGCT
MUC2	F: CAGGATACGTGTGTGCCCAT
	R: GGACGCGTTGCAATCAAAGT
GPX-1	F: CAATTCGGGCACCAGGAGAA
	R: GTACTGCGGGTTGGTCATCA
AvBD2	F: CTCTCTCCTCTTCCTGGCAC
	R: GAGGGGTCTTCTTGCTGCTG
AvBD4	F: ACGCTGATCTGCAGGACTAC
	R: GAGAACGGGAAAAGCCCACAG
Nrf2	F: GGCCACCCTAAAGCTCCATT
	R: GGCTTCACTGAACTGCTCCT
HO-1	F: ACAACGCTGAAAGCATGTCC
	R: GGATGCTTCTTGCCAACGAC
CAT	F: AGCTTGCAAAATGGCTGACG
	R: ATAGCCAAAGGCACCTGCTC
SOD-1	F: CCAAAAGATGCAGATAGGCACG
	R: GCAGTGTGGTCCGGTAAGAG
GCLM	F: CTGAGTCACGGTGTCGCTCC
	R: TCCAACCTTTGAGCTCCATTCA
GAPDH	F: AGTCGGAGTCAACGGATTTGG
	R: AAGATAGTGATGGCGTGCCC

### Western blotting analysis

Approximately 0.1 g of ileal tissue was homogenized in 1 mL of RIPA lysis buffer (Solarbio, R0010, Beijing, China) supplemented with protease and phosphatase inhibitor cocktails. After incubation on ice for 10 min, the lysates were centrifuged for 15 min at 4 °C, and the supernatant was collected. Protein concentrations were quantified using a BCA protein assay kit (Solarbio, Nanjing, China) following the manufacturer’s protocol, using a 96-well plate and standard curve.

Protein samples were mixed with 6 × SDS loading buffer at a ratio of 1:5 and denatured by heating at 100 °C for 5 min. Equal amounts of total protein (30 μg per well) were separated by SDS-PAGE and transferred onto polyvinylidene fluoride (PVDF) membranes (Millipore, United States), which were pre-activated in methanol. Membranes were blocked with 5% skim milk in TBST for 1 h at room temperature and incubated overnight at 4 °C with primary antibodies: anti-p-AKT (Rabbit polyclonal, WL03851, Wanleibio, 1:1000), anti-AKT (Rabbit polyclonal, WL0003, Wanleibio, 1:1000), anti-p-NF-κB (Rabbit polyclonal, WL02166, Wanleibio, 1:1000), anti-NF-κB (Rabbit polyclonal, WL01508, Wanleibio, 1:1000), and anti-GAPDH (Mouse monoclonal, Clone 6C5, WL01544a, Wanleibio, 1:1000). After washing three times with TBST (10 min each), membranes were incubated with HRP-conjugated goat anti-mouse IgG secondary antibody (WLA024, Wanleibio, 1:5000) for 1 h at room temperature. Immunoreactive bands were visualized using enhanced chemiluminescence (ECL) reagent (Wanleibio). Detailed antibody information is provided in Supplementary Table S1. Band intensities were analyzed using ImageJ software (12), antibodies were purchased from Abimat Pharmaceutical Technology Company.

### Statistical analysis

All experiments included triplicate biological replicates. Data are expressed as mean ± SD and analyzed using SPSS 26.0 (SPSS Inc., Chicago, IL, United States). Normality was verified via D’Agostino-Pearson and Kolmogorov–Smirnov tests. Normally distributed data with homogeneous variance underwent one-way ANOVA with Dunnett’s *post hoc* test; non-parametric data were analyzed by Kruskal–Wallis test with Dunn’s correction. Graphical representations were created using GraphPad Prism 8.0. Statistical significance was set at *p* < 0.05, and significance levels were denoted as follows: ns (not significant), ^*^p < 0.05, ^**^p < 0.01, and ^***^p < 0.001.

## Results

### Effect of dietary BCU on diarrhea rates and mortality

As detailed in Table 3, 73% of the chicks exhibited diarrhea, and 53% died after *E. coli* infection. In contrast, the diarrhea and mortality rates of chicks in BCU treatment groups were significantly lower. Notably, in the BCU40 group, the diarrhea rate decreased to 27% and the mortality rate to 7%, respectively. Suggested that BCU effectively mitigates the incidence of diarrhea and reduces mortality associated with *E. coli* disease.

**Table 3:** Diarrhea rate and mortality.

Group	Diarrhea rate (%)	Mortality (%)
Control	0	0
*E. coli*	73%	53%
BCU10	33%	33%
BCU20	40%	27%
BCU40	27%	7%

### Morphological observation of ileum tissue

**Figure 1:**
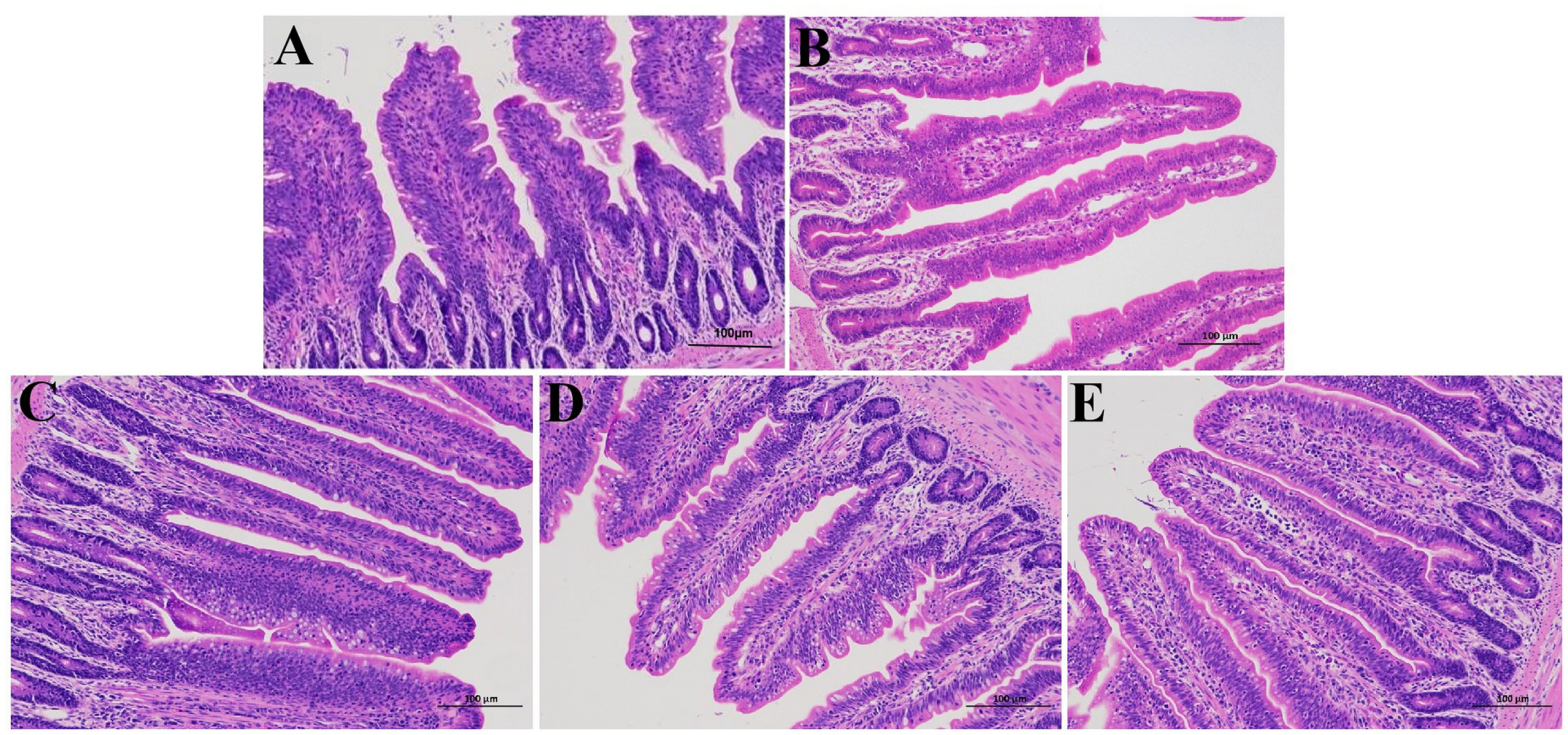
Effect of baicalin-copper complex on histopathological structure in chick ileum (scale bar = 100 μm). **(A)** control group; **(B)** *E. coli* group; **(C)** BCU10 (10 mg/kg BCU) group; **(D)** BCU20 (20 mg/kg BCU) group; **(E)** BCU40 (40 mg/kg B) group. In the images, the arrowheads indicate the location of the lesions **(B)**.

Figure 1 shows the results of histopathological observation of the ileum. In the control group, the villi structure of ileum was clear and complete, and columnar epithelial cells were closely connected. In contrast, APEC-infected chicks showed the proliferation of small intestinal crypts, villi atrophy, and obvious inflammatory cell infiltration and bleeding. In comparison with the *E. coli* group, the infiltration of inflammatory cells in ileum of chicks was reduced, and the bleeding was reduced in BCU treatment groups, and goblet cells increased significantly.

### Effect of BCU on mRNA expression of mucin and antimicrobial peptide in ileum induced by *Escherichia coli*

**Figure 2:**
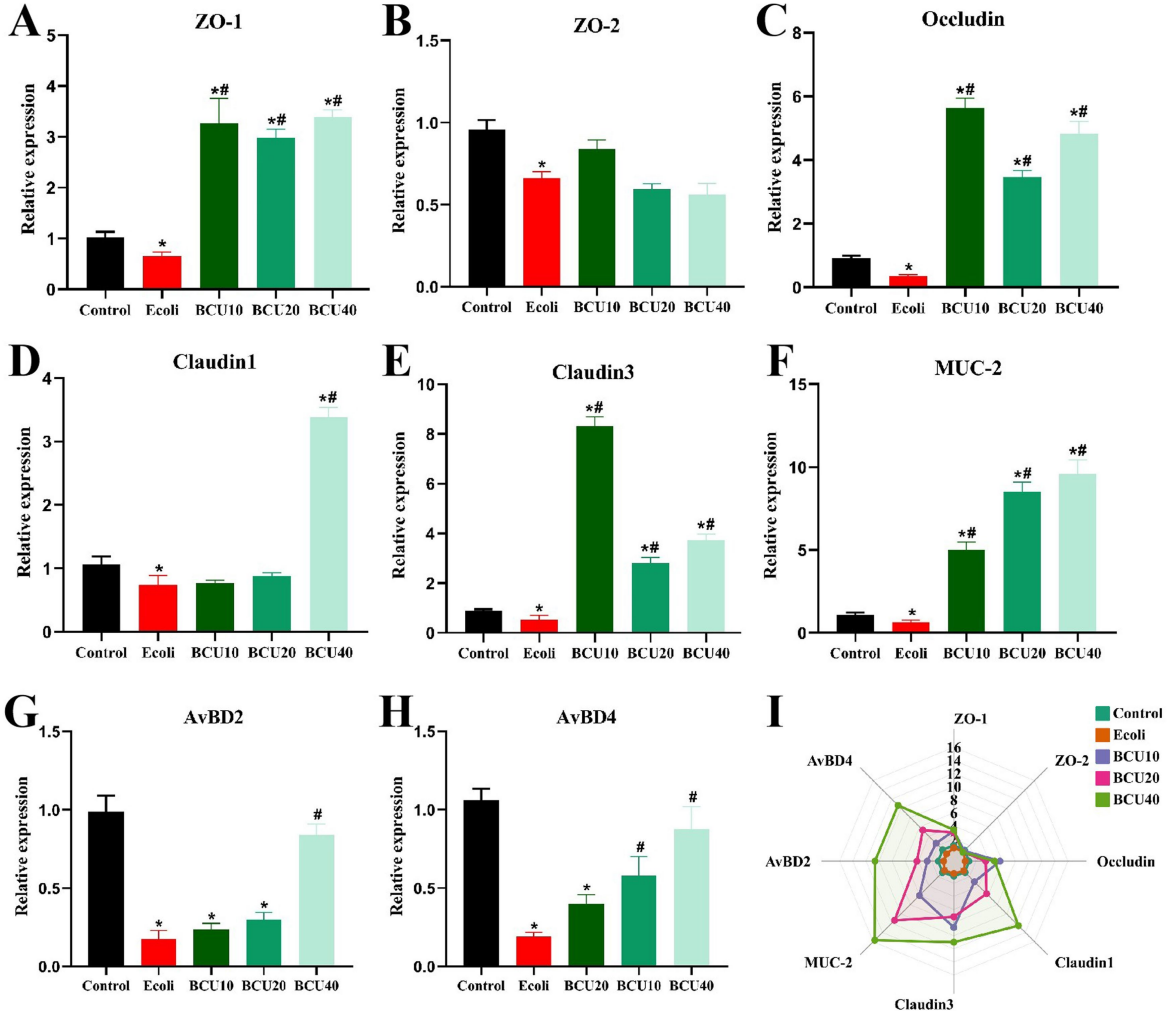
Effect of baicalin-copper complex on the mRNA expression of Mucosal Tight-Junction Proteins in ileum of chicks under the action of pathogenic *Escherichia coli*. **(A-H)**; **(I)** Radar chart shows the mRNA levels of intestinal barrier; Effects of the control, *E. coli*, BCU10, BCU20, and BCU40 groups on the mRNA levels of intestinal barrier genes. Data were represented as the mean ± SD (*n* ≥ 3 per group). Statistical analysis was performed using one-way ANOVA followed by Tukey’s post hoc test. “*” indicates a statistically significant difference between the control group and all other treatment groups (including the *E. coli*-infected group and BCU-treated groups). “#” indicates statistically significant difference between *E. coli* group and (model group) and the BCU treatment groups. *P < 0.05, **P < 0.01 vs. control group; ^#^*P* < 0.05, ^##^*P* < 0.01 vs. *E. coli*-infected (model) group.

As observed in Figure 2, the expression levels of intestinal barrier proteins ZO-1, ZO-2, occludin, claudin-1, claudin-3, MUC-2, AvBD2 and AvBD4 in *E. coli* group were significantly lower than those in control group (*p* < 0.05). In contrast to the *E. coli* group, the expression levels of ZO-1, occludin, claudin-3, and MUC-2 mRNAs were significantly increased in the BCU10, BCU20, and BCU40 groups (*p* < 0.05). Additionally, there was no significantly different in the expression levels of ZO-2 mRNA between BCU10, BCU20, BCU40 groups and *E. coli* group (*p* > 0.05). The expression of claudin-1 mRNA in BCU40 group was significantly higher than that in *E. coli* group (*p* < 0.05). In addition, compared with the control group, the mRNA expression levels of AvBD2 and AvBD4 were significantly decreased in the *E. coli* group (*p* < 0.05). However, both AvBD2 and AvBD4 mRNA levels were significantly up-regulated in the BCU40 group compared to the *E. coli* group (*p* < 0.05), and AvBD4 expression was also significantly elevated in the BCU20 group (*p* < 0.05).

### Effects of BCU on oxidative stress in ileum induced by *Escherichia coli*

**Figure 3:**
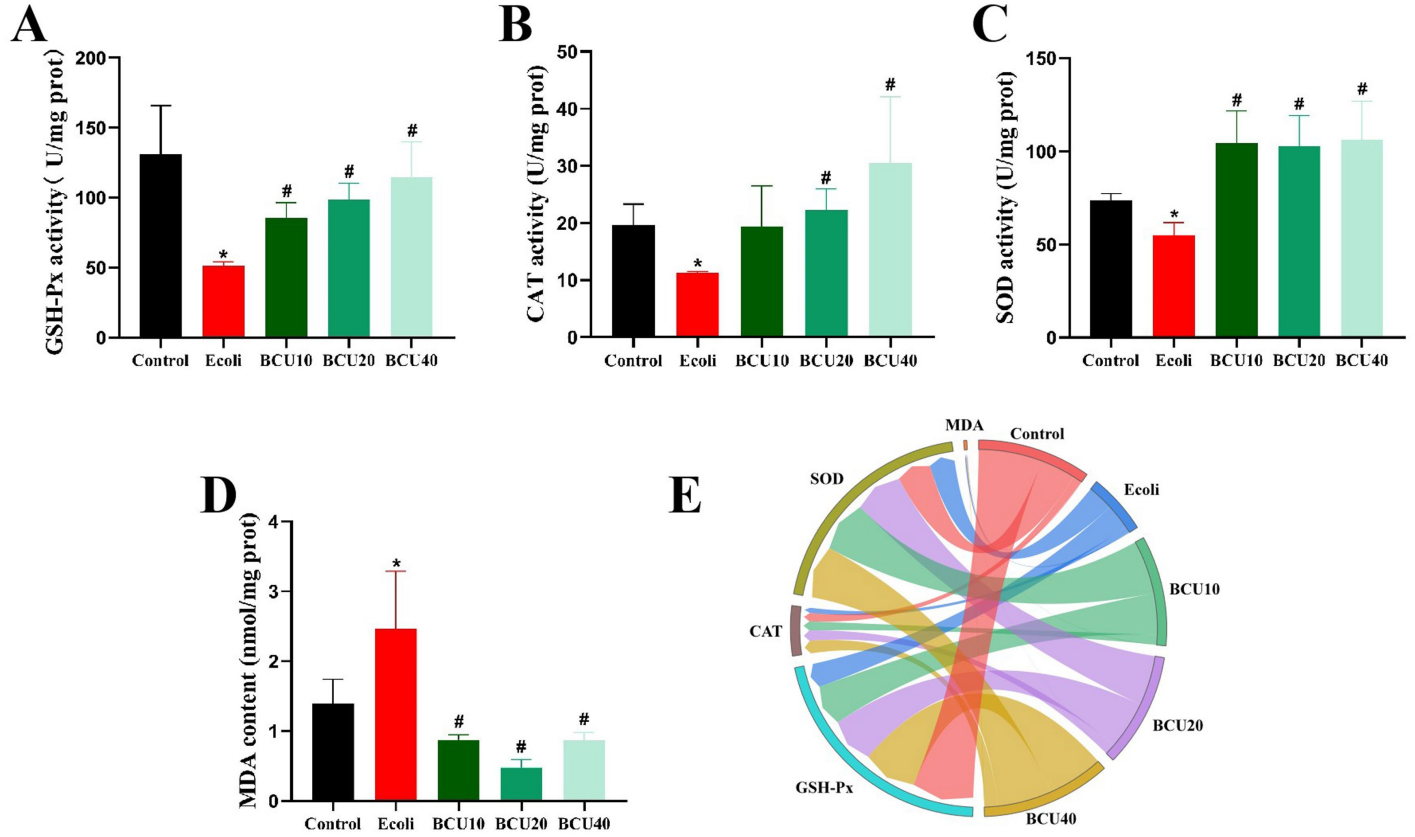
Effect of baicalin-copper complex on ileal antioxidant enzyme activity of chicks under the action of pathogenic *Escherichia coli*. **(A)** GSH-Px activity; **(B)** CAT activity; **(C)** SOD activity; **(D)** MDA content; **(E)** The chord chart of the expression of oxidative stress-related factors. Data were represented as the mean ± SD (*n* ≥ 3 per group). Statistical analysis was performed using one-way ANOVA followed by Tukey’s post hoc test. *P < 0.05, **P < 0.01 vs. control group; ^#^*P* < 0.05, ^##^*P* < 0.01 vs. *E. coli*-infected (model) group.

As identified in Figure 3, the results indicate that antioxidant enzyme activities (GSH-Px, CAT, SOD) reduced notably in *E. coli* group compare with control group. In contrast, the activities of these enzymes were significantly increased in the BCU groups compared with the *E. coli* group (*p <* 0.05). Furthermore, the levels of MDA were markedly elevated in *E. coli* group than in control group (*p* < 0.05). However, MDA levels were remarkably reduced in the BCU-supplemented groups in comparison to *E. coli* group (*p* < 0.05). In Figure 3E, the chord chart of the expression of oxidative stress-related factors is consistent with the above results.

### Effects of BCU on mRNA expression of antioxidant genes in ileum induced by *Escherichia coli*

**Figure 4:**
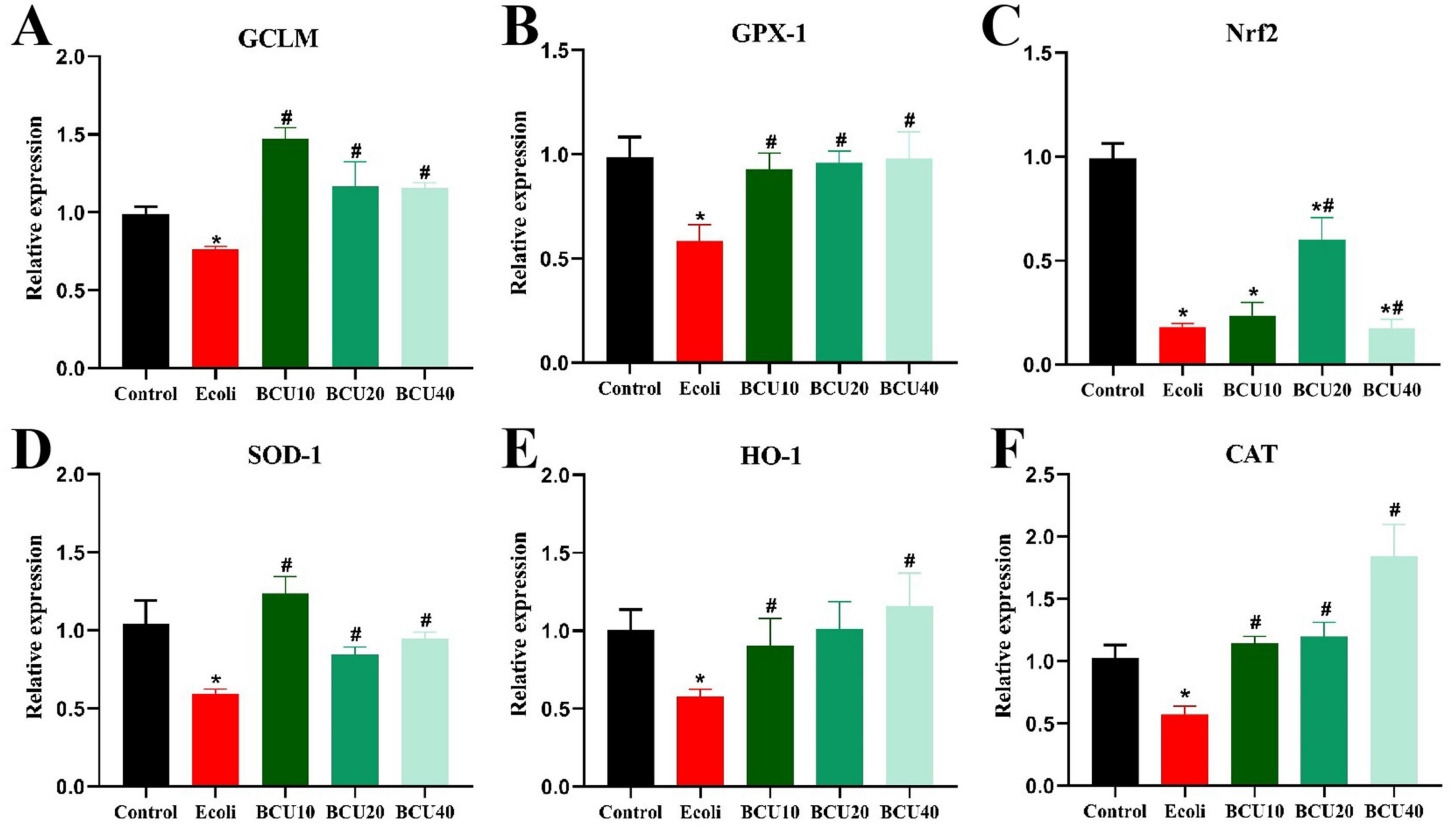
Effect of baicalin-copper complex on mRNA expression of antioxidant gene in ileum of chicks under the action of pathogenic *Escherichia coli*. **(A-F)** The mRNA levels of antioxidant (GCLM, CPX-1, Nrf2, SOD-1, HO-1 and CAT) genes. Data were represented as the mean ± SD (*n* ≥ 3 per group). Statistical analysis was performed using one-way ANOVA followed by Tukey’s post hoc test. *P < 0.05, **P < 0.01 vs. control group; ^#^ *P* < 0.05, ^##^*P* < 0.01 vs. *E. coli*-infected (model) group.

As illustrated in Figure 4, compared with control group, the expression levels of antioxidant genes GCLM, GPX-1, SOD-1, Nrf-2, HO-1, and CAT mRNA in *E. coli* group showed significantly decreased (*p* < 0.05). In contrast, mRNA expression of the above genes was significantly increased (*p* < 0.05) in the BCU-supplemented group compared with the *E. coli* group. The findings revealed that BCU upregulates the mRNA expression of antioxidant genes in the intestinal tract of chicks, thereby mitigating oxidative stress damage caused by avian *E. coli* infection.

### Effects of BCU on mRNA expression of inflammatory factor in ileum induced by *Escherichia coli*

**Figure 5:**
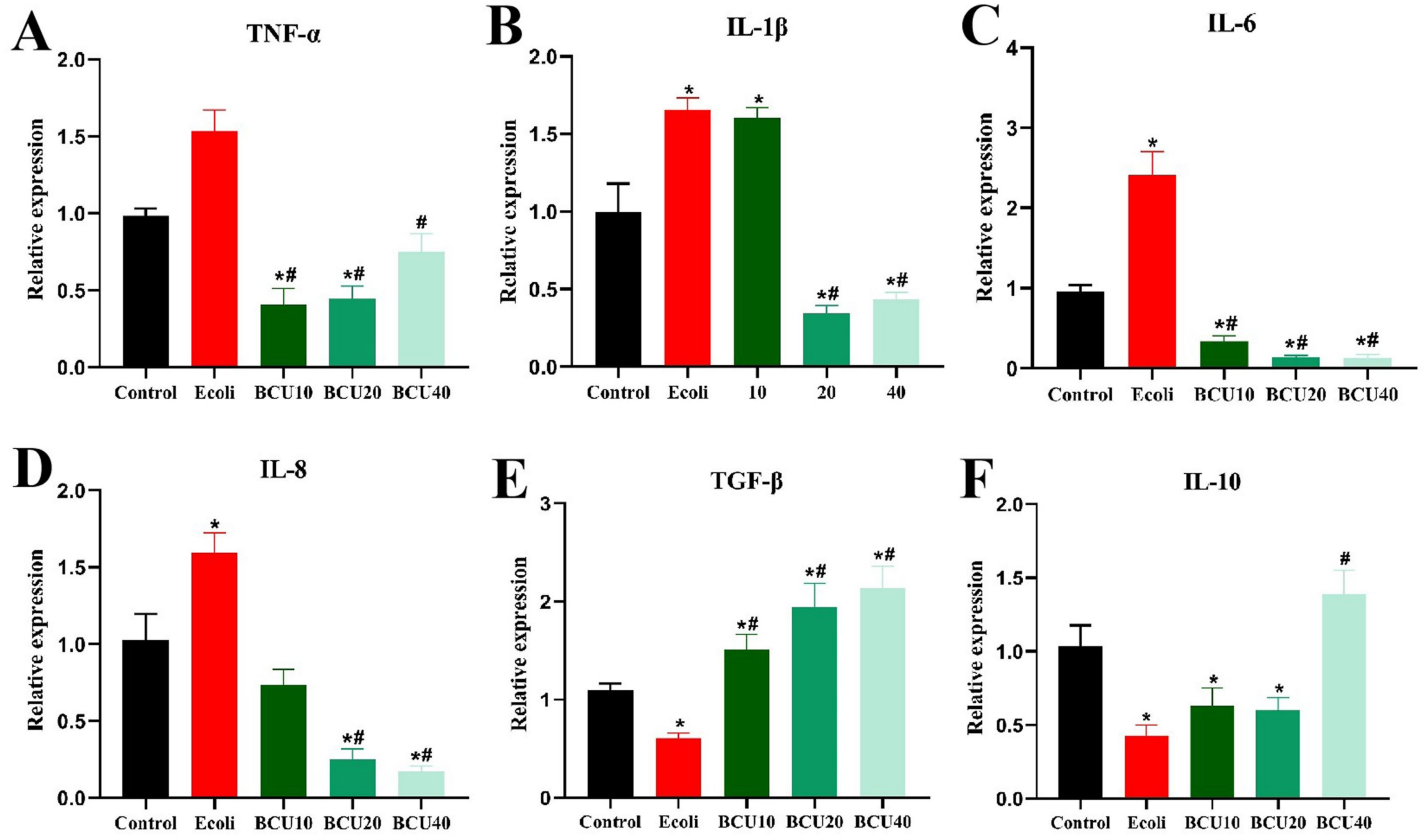
Effect of baicalin-copper complex on mRNA expression of Inflammatory factors mRNA in ileum of chicks under the action of pathogenic *Escherichia coli*. **(A-D)** The mRNA levels of proinflammatory (TNF-α, IL-1β, IL-6 and IL-8) genes; **(E-F)** The mRNA levels of anti-inflammatory (TNF-β and IL-10). Data were represented as the mean ± SD (*n* ≥ 3 per group). Statistical analysis was performed using one-way ANOVA followed by Tukey’s post hoc test. *P < 0.05, **P < 0.01 vs. control group; ^#^*P* < 0.05, ^##^*P* < 0.01 vs. *E. coli*-infected (model) group.

As shown in Figure 5, compared to the control group, the mRNA expressions of inflammatory factors TNF-α, IL-1β, IL-6 and IL-8 in the ileum of *E. coli* group showed a significant increase (*p* < 0.05). In contrast, the expression level of inflammatory factors mRNA in BCU-supplemented group was significantly lower than that in *E. coli* group (*p* < 0.05). Anti-inflammatory factor TGF-β and IL-10 mRNA expression levels were notably lower in *E. coli* group than those in control group (*p* < 0.05). The expression level of anti-inflammatory factor TGF-β mRNA in the ileum of chicks add with BCU was markedly higher than that in *E. coli* group (*p* < 0.05). Meanwhile, the expression of the anti-inflammatory factor IL-10 mRNA was also significantly higher in the ileum of chicks in the BCU40 group, which was significantly higher than that in *E. coli* group (*p* < 0.05).

### Effects of BCU on the expression of NF-ΚB and AKT proteins in ileum induced by *Escherichia coli*

**Figure 6:**
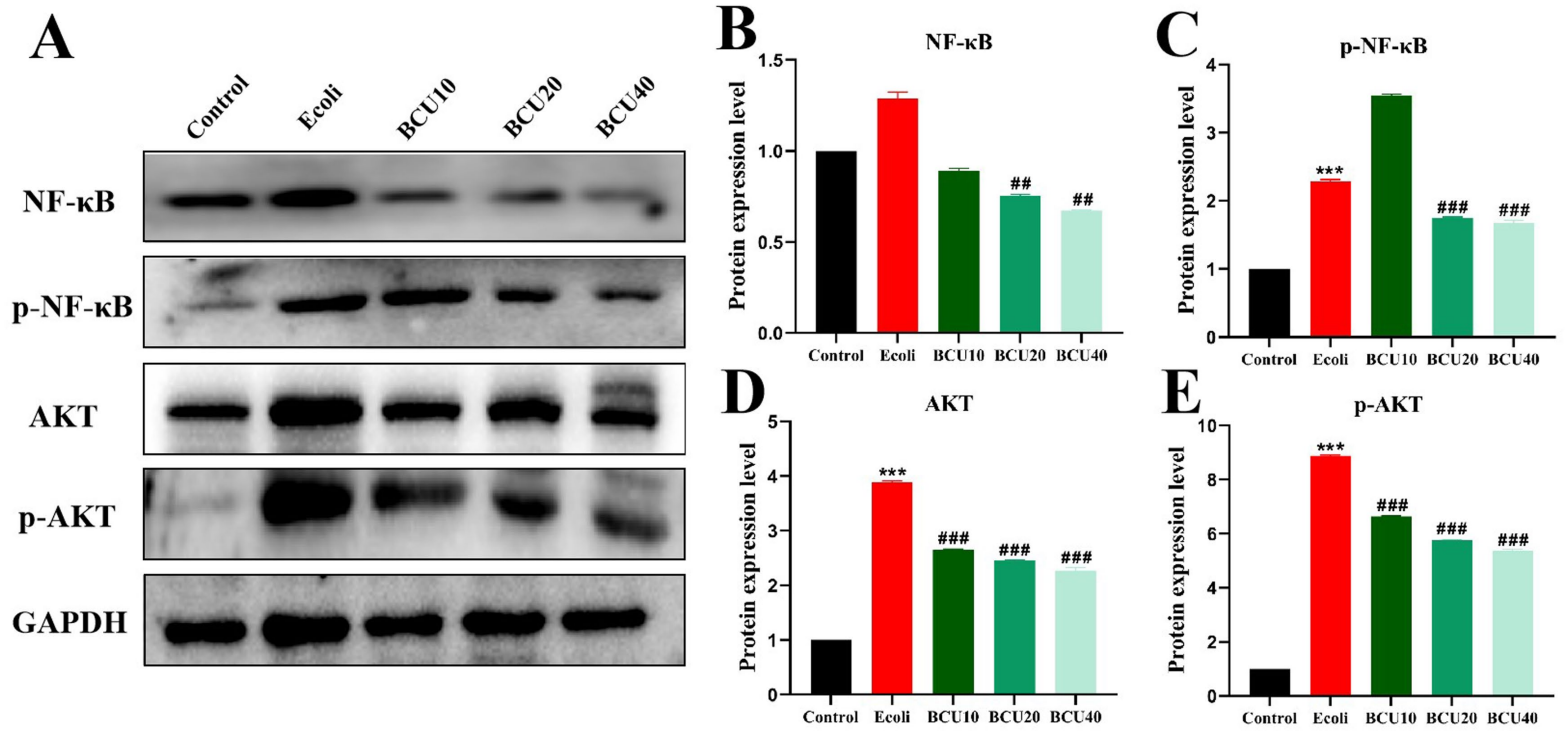
Effect of baicalin-copper complex on NF-κB and AKT proteins in ileum of chicks induced by *Escherichia coli*. **(A-E)** The protein levels of NF-kB, p-NF-kB, AKT, p-AKT. Data were represented as the mean ± SD (*n* ≥ 3 per group). Statistical analysis was performed using one-way ANOVA followed by Tukey’s post hoc test. *P < 0.05, **P < 0.01 vs. control group; ^#^*P* < 0.05, ^##^*P* < 0.01 vs. *E. coli*-infected (model) group.

The results were described in Figure 6, compared with the control group, the protein expression levels of NF-κB, AKT, p-NF-κB and p-AKT in *E. coli* group increased. The results of BCU20 and BCU40 groups showed that the expression levels of p-NF-κB and p-AKT were significantly lower than those of *E. coli* group (*p* < 0.05), and the level of p-AKT protein in BCU 10 group was also significantly lower. The above results indicated that BCU can effectively regulate inflammation-related protein expression in chick ileum after avian *E. coli* infection, thus alleviating the inflammatory damage in the intestinal tract of chicks caused by avian *E. coli*.

### The abundance and correlation analysis of mRNA levels

**Figure 7:**
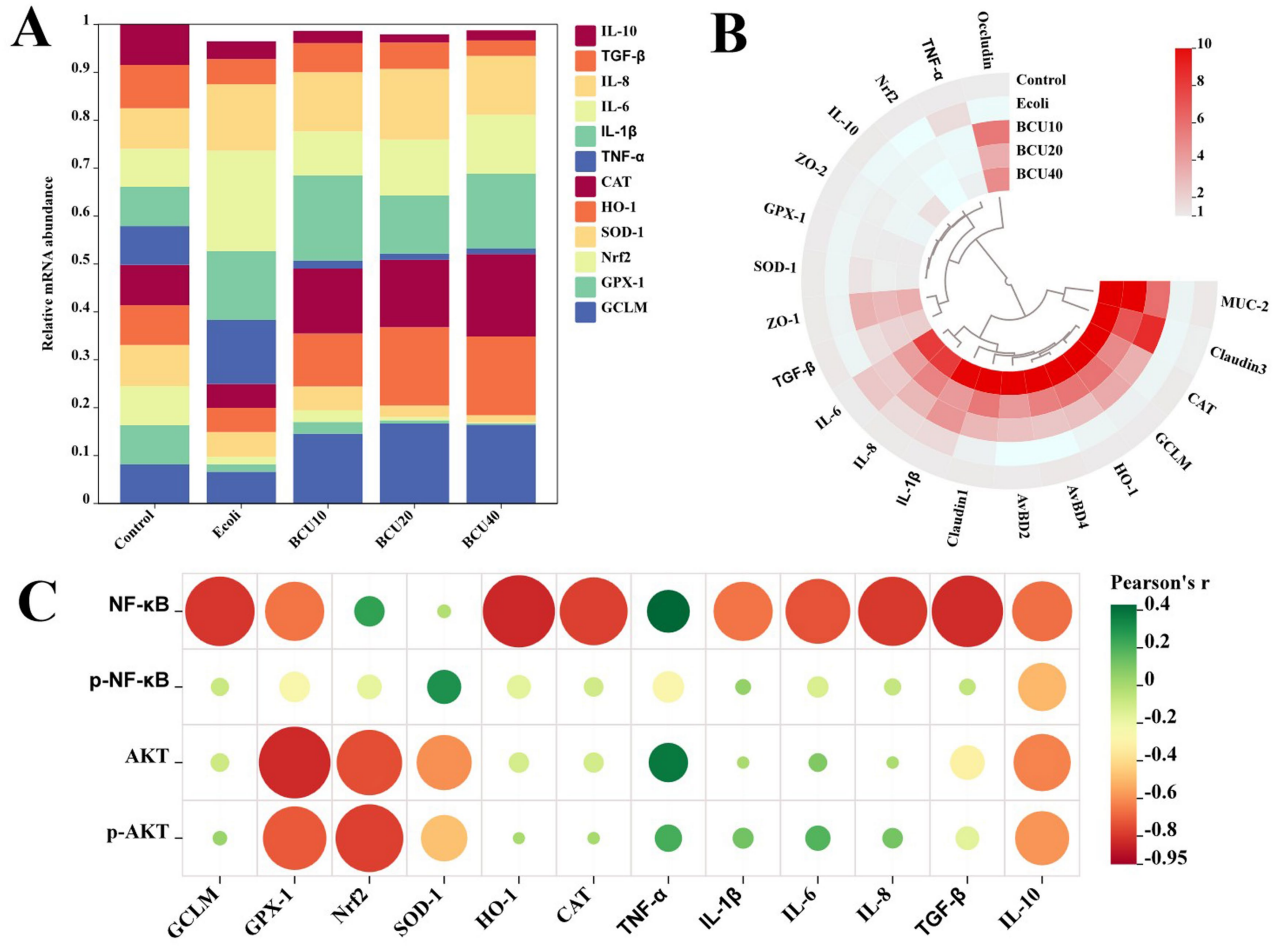
The correlative analysis of mRNA levels. **(A)** The abundance of mRNA in each group. **(B)** The circle heatmap analysis of mRNA levels. **(C)** The correlative analysis between the expression levels of AKT/NF-κB pathway-related proteins and mRNA levels of inflammation-and oxidative stress-related factors.

As described in Figure 7, variations existed in mRNA abundance across different groups. In addition, there was also a positive correlation between APEC treatment and mRNA levels of antioxidants, inflammatory factors and intestinal tight junction proteins, but a negative association with the mRNA levels of anti-inflammatory factors. Meanwhile, AKT/NF-κB pathway protein levels were positively associated with mRNA levels of factors related to antioxidants and inflammation, but negatively associated with mRNA levels of factors related to anti-inflammatory.

## Discussion

Avian colibacillosis is a widespread and highly contagious disease affecting chickens of all ages, with chicks being especially susceptible. Infected chicks often display intestinal damage, including mucosal edema, hemorrhage, and epithelial disruption (13, 14). When the intestinal epithelial barrier is compromised, translocation of luminal bacteria and endotoxins (e.g., lipopolysaccharides) into the bloodstream can occur, triggering systemic inflammatory responses. Moreover, tight junction disruption impairs nutrient absorption by altering epithelial integrity and transporter function. Given that intestinal barrier compromise results in low disease resistance and decreased production performance in chicks, maintaining its integrity is vital. Recent studies have explored natural compounds with both antibacterial and antitoxin properties for preventing such damage (15, 16). BCU, a complex of baicalin and copper ions, is known for its antibacterial, antioxidant, and immunomodulatory activities (17, 18). Meanwhile, Zhao et al. (19) demonstrated that BCU significantly attenuates *E. coli*-induced sepsis by reducing bacterial virulence and suppressing host inflammatory responses. Chen et al. (20) highlighted that BCU exerts its antibacterial activity primarily through membrane disruption, leading to leakage of cellular contents and bacterial death. These findings collectively support the dual function of BCU in both direct bacterial killing and modulation of the host immune response. This study examined the effects of BCU at various concentrations on chicks challenged with APEC. APEC infection alone resulted in a 73% diarrhea rate and 53% mortality, while BCU supplementation significantly reduced both. Histopathological analysis further confirmed that BCU mitigated intestinal damage by alleviating villous atrophy, hemorrhage, and inflammation.

The intestinal epithelial barrier plays a crucial role in limiting systemic inflammation and maintaining homeostasis (21). Barrier dysfunction not only permits microbial translocation and endotoxin entry into systemic circulation, but also impairs nutrient absorption efficiency, collectively exacerbating systemic inflammation and metabolic burden (22, 23). Intestinal injury typically arises from mucosal damage and increased permeability. Tight junctions (TJs) comprise transmembrane and scaffold proteins such as occludin, claudins, and zonula occludens (ZO-1, ZO-2), which link to the cytoskeleton and regulate paracellular permeability (24, 25). The coordinated assembly of these proteins forms a dynamic mechanical barrier that responds to external insults (26, 27). Additionally, the chemical barrier consists of mucus secreted by goblet cells, primarily composed of mucin 2 (MUC2), and is fortified by antimicrobial peptides like avian β-defensins (AvBD2, AvBD4) and secretory IgA, which collectively maintain intestinal defense and immune exclusion, which collectively prevent pathogen adherence (28, 29). The experiment results showed that APEC infection reduced the ileal expression level of key barrier components, including ZO-1, ZO-2, occludin, claudin-1, claudin-3, MUC2, AvBD2, and AvBD4, suggesting that the intestinal barrier was compromised, in line with research by Wu et al. (30). Conversely, the levels of these genes mRNA were found to be notably up-regulated in response to BCU treatment. These results suggested that APEC affected the intestinal health of chicks by destroying the intestinal barrier and causing intestinal damage, whereas BCU could markedly improve these conditions.

Prior studies demonstrated that BCU exerts antibacterial effects through disrupting bacterial cell membrane integrity and inducing oxidative damage via copper-mediated Fenton-like reactions (31). The BCU can generate reactive oxygen species (ROS) through redox cycling of Cu^2+^/Cu^+^, leading to oxidative damage to bacterial membranes, proteins, and nucleic acids, especially in Gram-negative pathogens (32). This dual action underlies its broad-spectrum antibacterial potential (33), highlighting its potential as a dual-function therapeutic. It also possesses antioxidant properties, helping neutralize free radicals and reduce oxidative stress-related tissue injury (34). SOD, CAT, and GSH-Px are essential antioxidant enzymes that constitute the primary defense mechanism against reactive oxygen species (ROS). Meanwhile, MDA, a secondary product of lipid peroxidation, is commonly used as a marker to assess oxidative damage in tissues (35). In this study, APEC infection significantly decreased the activities of SOD, CAT, and GSH-Px, while increasing MDA levels, indicating oxidative damage. Moreover, the expression of antioxidant genes (GCLM, GPX-1, SOD-1, Nrf2, HO-1, and CAT) in the ileum was down-regulated. However, BCU administration reversed these effects, enhancing antioxidant enzyme activities and gene expression, and reducing MDA accumulation. These findings suggest that BCU alleviates APEC-induced oxidative stress and preserves intestinal redox balance, which may help prevent further inflammation.

Inflammation is a fundamental host response to microbial invasion, primarily mediated by pro-inflammatory cytokines. Among them, TNF-α plays a central role by activating the NF-κB signaling pathway, amplifying inflammatory responses. In contrast, IL-10 and TGF-β help restrain excessive inflammation (36, 37). In this study, APEC infection elevated pro-inflammatory cytokine expression in the ileum, while BCU administration significantly suppressed these cytokines and up-regulated IL-10 and TGF-β levels, suggesting a protective anti-inflammatory effect. Mechanistically, AKT activation is known to promote NF-κB signaling through phosphorylation cascades (38). Our data revealed that APEC markedly increased p-AKT and p-NF-κB levels, indicating pathway activation. However, BCU treatment attenuated this response, downregulating both phosphorylated proteins. These findings suggest that BCU may exert its anti-inflammatory action by inhibiting AKT/NF-κB signaling. Collectively, BCU alleviated intestinal injury in APEC-infected chicks by reducing inflammation, oxidative damage, and epithelial barrier disruption, which demonstrated that BCU may represent a potential therapeutic strategy for the treatment of inflammatory diseases associated with *E. coli*. These findings provide compelling evidence for the application of phytochemicals in poultry health management (39).

## Conclusion

In summary, infection with avian *E. coli* induces morphological damage to the ileum in chicks, leading to oxidative stress and inflammatory responses. Additionally, BCU effectively mitigates *E. coli*-induced damage to the intestinal tract, oxidative stress, and inflammation in chicks. This protective effect is correlated with the modulation of the AKT/NF-κB signal pathway.

## Data Availability

The original contributions presented in the study are included in the article/Supplementary material, further inquiries can be directed to the corresponding author.
