# Supplementary material for: Baicalin-copper complex alleviates intestinal damage in avian pathogenic Escherichia coli-infected chicks by targeting the AKT/NF-κB pathway
Source: Front Vet Sci. 2025 Aug 26;12:1648736. doi: 10.3389/fvets.2025.1648736 (PMC12418448; doi:10.3389/fvets.2025.1648736)
Supplement: Supplementary file 1 [file Table_1.docx]

**Table S1**. List of primary and secondary antibodies used in this study

| **Target Protein** | **Host Species** | **Clone** | **Dilution** | **Catalog Number** | **Supplier** |
| --- | --- | --- | --- | --- | --- |
| P-AKT | Rabbit | Polyclonal | 1:1000 | WL03851 | Wanleibio |
| AKT | Rabbit | Polyclonal | 1:1000 | WL0003 | Wanleibio |
| P-NF-κB | Rabbit | Polyclonal | 1:1000 | WL02166 | Wanleibio |
| NF-κB | Rabbit | Polyclonal | 1:1000 | WL01508 | Wanleibio |
| GAPDH | Mouse | Monoclonal | 1:1000 | WL01544a | Wanleibio |
| HRP Goat Anti-Rabbit IgG | Goat | - | 1:5000 | WLA023 | Wanleibio |
| HRP Goat Anti-Mouse IgG | Goat | - | 1:5000 | WLA024 | Wanleibio |
